# Flow Cytometry Detection of Anthracycline-Treated Breast Cancer Cells: An Optimized Protocol

**DOI:** 10.3390/cimb45010013

**Published:** 2022-12-28

**Authors:** Giulia Catitti, Simone De Fabritiis, Davide Brocco, Pasquale Simeone, Domenico De Bellis, Simone Vespa, Serena Veschi, Laura De Lellis, Nicola Tinari, Fabio Verginelli, Marco Marchisio, Alessandro Cama, Antonia Patruno, Paola Lanuti

**Affiliations:** 1 Department of Medicine and Aging Sciences, University “G. d’Annunzio” of Chieti-Pescara, 66100 Chieti, Italy; 2 Center for Advanced Studies and Technology (CAST), University “G. d’Annunzio” of Chieti-Pescara, 66100 Chieti, Italy; 3 Department of Neurology, Karolinska University Hospital, 17177 Stockholm, Sweden; 4 Department of Pharmacy, University “G. d’Annunzio” of Chieti-Pescara, 66100 Chieti, Italy; 5 Department of Medical, Oral & Biotechnological Sciences, University “G. d’Annunzio” Chieti-Pescara, 66100 Chieti, Italy

**Keywords:** polychromatic flow cytometry, anthracycline autofluorescence, apoptosis, breast cancer cells

## Abstract

The use of anthracycline derivatives was approved for the treatment of a broad spectrum of human tumors (i.e., breast cancer). The need to test these drugs on cancer models has pushed the basic research to apply many types of in vitro assays, and, among them, the study of anthracycline-induced apoptosis was mainly based on the application of flow cytometry protocols. However, the chemical structure of anthracycline derivatives gives them a strong autofluorescence effect that must be considered when flow cytometry is used. Unfortunately, the guidelines on the analysis of anthracycline effects through flow cytometry are lacking. Therefore, in this study, we optimized the flow cytometry detection of doxorubicin and epirubicin-treated breast cancer cells. Their autofluorescence was assessed both by using conventional and imaging flow cytometry; we found that all the channels excited by the 488 nm laser were affected. Anthracycline-induced apoptosis was then measured via flow cytometry using the optimized setting. Consequently, we established a set of recommendations that enable the development of optimized flow cytometry settings when the in vitro assays of anthracycline effects are analyzed, with the final aim to reveal a new perspective on the use of those in vitro tests for the further implementation of precision medicine strategies in cancer.

## 1. Introduction

The recently increasing interest in cell-specific drugs has pushed basic research to explore many types of in vitro tests. Cytotoxicity evaluation, the assessment of cell cycle analysis, the study of apoptosis induction, and cell–cell interaction both at molecular and biochemical levels are among the most widely applied in vitro assays to test cell-specific drug effects [1,2,3,4,5]. The aim of these in vitro studies is the discovery of novel drugs, including effective cancer cell-specific agents, to further test in preclinical models [3,6,7,8,9,10,11]. In this regard, anthracycline derivatives are among the most used cytotoxic drugs to treat human malignancies and have been mainly investigated in preclinical studies [12,13]. Considering their cornerstone role in many approved anticancer treatment regimens, novel in vitro approaches based on testing anthracycline derivatives may result in innovative clinical applications [14,15,16]. 

Doxorubicin (adriamycin) is one of the most powerful drugs among anthracyclines, but its use has been limited by the known doxorubicin-induced fatal cardiotoxic events [17]. To overcome such a limitation, different anthracycline analogues have been synthesized. Among them, its semisynthetic C-4′ sugar epimer epirubicin gained clinical interest, due to having similar antitumor efficacy to doxorubicin but with a lower risk of cardiotoxicity [18]. Overall, anthracycline derivatives are typically characterized by a planar oxidized anthracene nucleus fused to a cyclohexane ring that is connected to an amino sugar via a glycosidic linkage. Anthracyclines act as cytostatic/cytotoxic agents by interacting with topoisomerase II and inducing an irreversible DNA breakdown [19]. It has been also reported that anthracycline derivatives inhibit DNA and RNA synthesis by intercalating between nitrogenous base pairs [19]. The additional mechanisms of action contributing to the antiproliferative effects of anthracyclines include the enhancement of synthesis of reactive oxygen species and the formation of DNA adducts [19,20].

Anthracycline-induced apoptosis has been largely investigated in different cancer models [21]. Many classical apoptotic hallmarks can be rapidly analyzed using flow cytometry. The most widely used flow cytometry method for identifying apoptotic cells is based on the detection of phosphatidylserine exposure on the outer leaflet of the cell plasma membrane [9,22,23,24]. Notably, anthracycline derivatives are characterized by a typical red fluorescence emission associated with their conjugate systems [25]. Doxorubicin has a maximum excitation and emission wavelength at 470 and 560 nm, respectively [26], while epirubicin has an excitation peak at 254 nm and an emission peak at 565 nm [27]. Therefore, anthracycline-induced cell autofluorescence must be considered when those drugs are analyzed with in vitro assays, especially when fluorescence-based techniques are used [28]. 

The guidelines on the analysis of anthracycline derivative effects using flow cytometry are lacking, and the reported data are often difficult to interpret. Thus, we developed a flow cytometry assay to optimize the study of anthracycline-induced apoptosis in breast cancer cells. Considering the autofluorescence associated with the concentrations of doxorubicin and epirubicin commonly employed for in vitro breast cancer cell studies, we underlined all the recommendations for an optimized flow cytometry analysis.

## 2. Materials and Methods

### 2.1. Cell Cultures

MDA-MB-231 human breast cancer cells (ATCC, Manassas, VA, USA) were maintained in a humidified atmosphere (5% CO_2_ at 37 °C), in Dulbecco’s modified Eagle medium (DMEM, ThermoFisher Scientific, Gibco; Waltham, MA, USA) containing high glucose concentration (4.5 g/l, or 25 mM) and supplemented with 10% fetal bovine serum (Merck KGaA, Darmstadt, Germania), 50 units/mL penicillin, and 50 mg/mL streptomycin (Merck). All the experiments were carried out on exponentially growing cells. 

MDA-MB-231 cells, seeded at a density of 2 × 10^5^ in six-well plates (Falcon, Corning Incorporated, One Riverfront Plaza, NY, USA, 353046) were treated for 24 h and 48 h with 2.5 μM doxorubicin, and 1 μM and 2.5 μM epirubicin. The anthracycline concentrations were established on the basis of previously published data [29]. In detail, we used the above-reported doxorubicin and epirubicin concentrations, which are slightly lower than the respective IC50, with the aim to focus on the effect related to the autofluorescence of the anthracycline chromophore groups. 

### 2.2. Flow Cytometry Analysis of Apoptosis

For apoptosis measurements, the Annexin V test was used. Annexin V is a calcium-dependent phospholipid-binding protein with a high affinity for phosphatidylserine (PS), a plasma membrane phospholipid. PS is physiologically exposed to the inner leaflet of the plasma membrane, but, during the earliest apoptosis phases, it translocates to the outer leaflet, thereby exposing PS to the external environment. Annexin V binds to the PS exposed on the cell surface and identifies apoptotic cells already during the earliest apoptotic phases. Here, the apoptosis was assessed as previously described [3]. Briefly, MDA-MB-231 cells were counted with a Burker chamber and seeded in 6-well plates (2 × 10^5^ cells/well) in triplicate. The following day, the cells were treated with 2.5 µM doxorubicin for 24 or 48 h. After treatment, apoptosis was measured by saving the supernatants before detaching the cells through trypsinization and washed once in PBS (ThermoFisher Scientific, Gibco; Waltham, MA, USA, 400 g, 10 min). The supernatant was discarded, and the cell pellet was resuspended at a concentration of 5 × 10^5^ cells/mL in Binding Buffer 1X (Becton Dickinson (BD) Biosciences, La Jolla, CA, USA). The samples were centrifuged (400 g, 10 min) and then stained using 5 μL of Annexin V-BV450 (BD Biosciences, Cod. 560506, 15 min, RT, in the dark). Before the acquisition, 300 μL of Binding Buffer 1X was added. For each sample, 20,000 events were acquired using a FACSVerse flow cytometer (BD Biosciences). Apoptotic cells were identified for their positivity to Annexin V, as shown in the gating strategy represented in Figure S1.

### 2.3. Flow Cytometry Measurements of Anthracycline Autofluorescence 

Doxorubicin- and epirubicin-treated MDA-MB-231 cells were analyzed for their intrinsic fluorescence, acquiring 20,000 events/test using different flow cytometry platforms (FACSCanto II, FACSVerse—both from BD Biosciences; CytoFLEX—Beckman Coulter, Fullerton, CA, USA) as well as an AMINS ImageStream (Luminex Corporation, Austin, TX), equipped with a 488 nm solid-state laser (40 mW) and Inspire software (v 4.1.434.0) [6].

To obtain comparable results, flow cytometry analyses were standardized by daily running quality controls, including check-ups with Cytometer Setup and Tracking Beads (CS&T, BD). Debris and doublets were excluded from the analysis, and single events were analyzed for different purposes. Optimal photomultiplier (PMT) voltages were established for each channel [30,31]. FACSDiva v 6.1.3 and FACSSuite v 1.0.6.5230 were used for data acquisition, and FlowJo v 10.8.1 Software (BD Biosciences) was used for data analysis.

### 2.4. Statistics

Statistical analysis was carried out using GraphPad Prism 9 (GraphPad Software, San Diego, CA, USA). Statistical significance was assessed (*p* < 0.05), and the standard error of the mean (SEM) was calculated for both the unstimulated and doxorubicin-stimulated samples.

## 3. Results

### 3.1. Impact of Doxorubicin Autofluorescence in Flow Cytometry Analyses 

**Figure 1:**
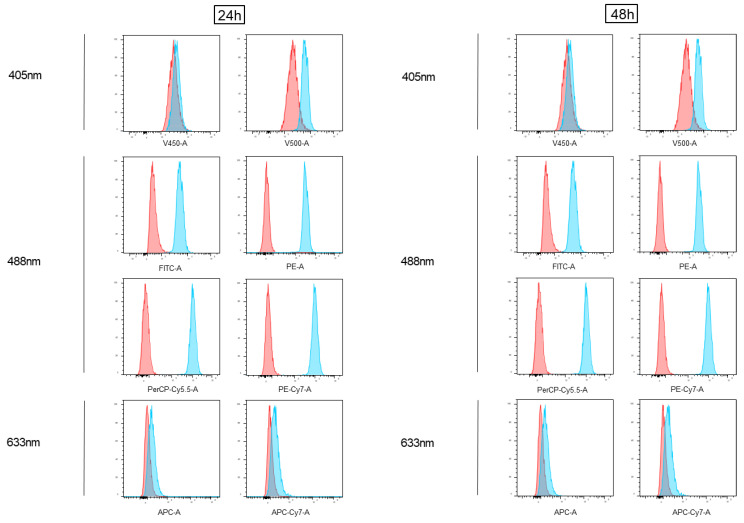
Analysis of doxorubicin fluorescence emission via conventional flow cytometry (BD FACSVerse). Cells treated with 2.5 µM doxorubicin (Doxo) for 24 and 48 h were acquired using flow cytometry and shown as blue histograms on every channel of a conventional instrument (FACSVerse, BD Biosciences), equipped with three lasers (488 nm, 633 nm, and 405 nm). Overlayed red histograms show the profiles of matched untreated samples. Histograms are representative of three independent experiments.

MDA-MB-231 cells were treated with doxorubicin for 24 and 48 h (DOXO) and analyzed using different flow cytometry conventional platforms (Figure 1, Figures S1 and S2). Specifically, as shown in Figure 1, the doxorubicin-treated cells were analyzed on each fluorescence channel (blue histograms) of a FACSVerse flow cytometer and compared with the untreated samples (overlayed red histograms) both at 24 and at 48 h. As shown (Figure 1), the doxorubicin-treated MDA-MB-231 cells showed detectable fluorescence levels in all the channels excited by the 488 nm laser and a slight signal in the farthest 405 nm excited channel.

Consistent results were obtained when the treated samples were acquired using other conventional flow cytometry platforms, such as a BD FACSCanto II (Figure S1) and a BC CytoFLEX (Figure S2). 

### 3.2. Impact of Epirubicin Autofluorescence in Flow Cytometry Analyses 

**Figure 2:**
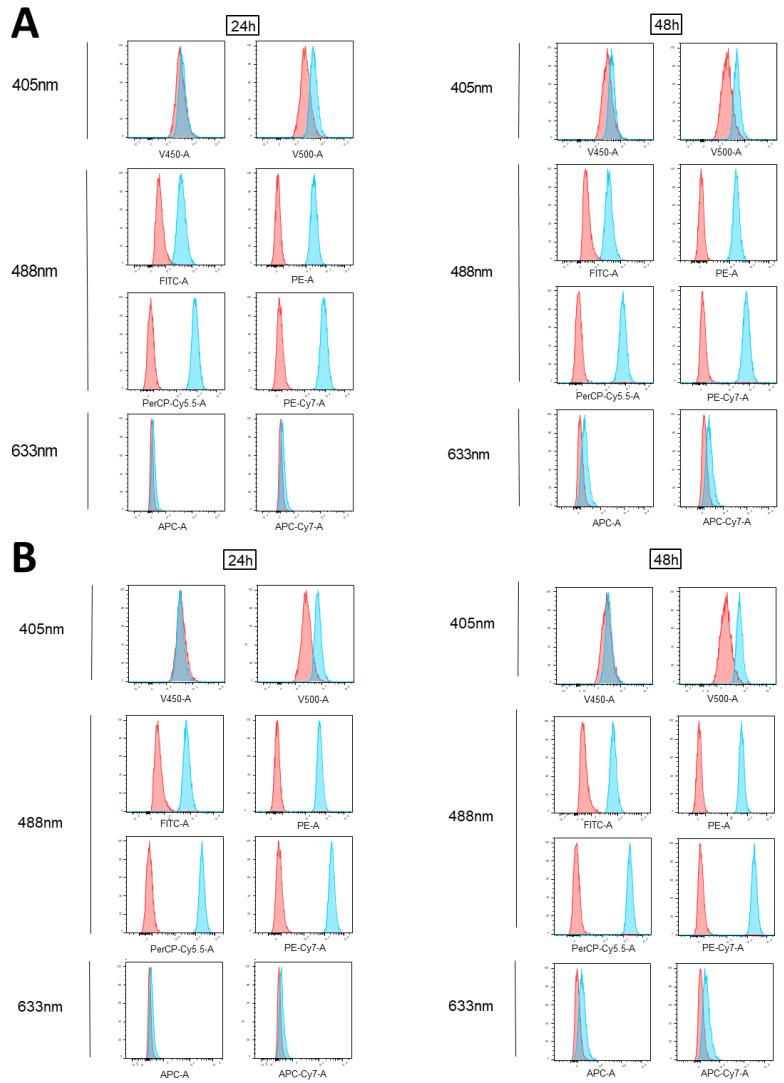
Analysis of epirubicin fluorescence emission via conventional flow cytometry (BD FACSVerse). MDA-MB-231 cells treated for 24 and 48 h with 1 µM (**A**) or 2.5 µM (**B**) epirubicin were acquired using flow cytometry and shown as blue histograms on every channel of a conventional instrument (FACSVerse, BD Biosciences), equipped with three lasers (488 nm, 633 nm, and 405 nm). Overlayed red histograms show the related untreated samples. Histograms are representative of three independent experiments.

Notably, MDA-MB231 cells were also treated with epirubicin, another anthracycline derivative, at 1 µM and 2.5 µM for 24 and 48 h. As shown in Figure 2, the epirubicin-treated MDA-MB-231 cells showed detectable fluorescence levels in all the channels excited by the blue laser (488 nm), at both concentrations. In addition, a slight signal in the farthest 405 nm excited channel was also detected. These results are consistent with those obtained by treating the cells with its analogue, doxorubicin.

When the same samples were acquired using other flow cytometry conventional platforms, a BD FACSCanto II and a Beckman Coulter CytoFLEX (Figures S4 and S5), the channels excited by the 488 nm laser appeared largely affected by the autofluorescence of this drug. 

### 3.3. Flow Cytometry Anthracycline Autofluorescence Is Dose-Dependent

We observed that the epirubicin autofluorescence contribution in each affected channel increased in a dose-dependent manner (Figure 2, Figures S4 and S5).

The signal-to-noise ratio (SNR) values were calculated for both drugs and reported for all the channels (Table 1). The data showed that the emission peak was registered at the PerCP-Cy5.5/PC5.5 channel for both drugs.

**Figure 3:**
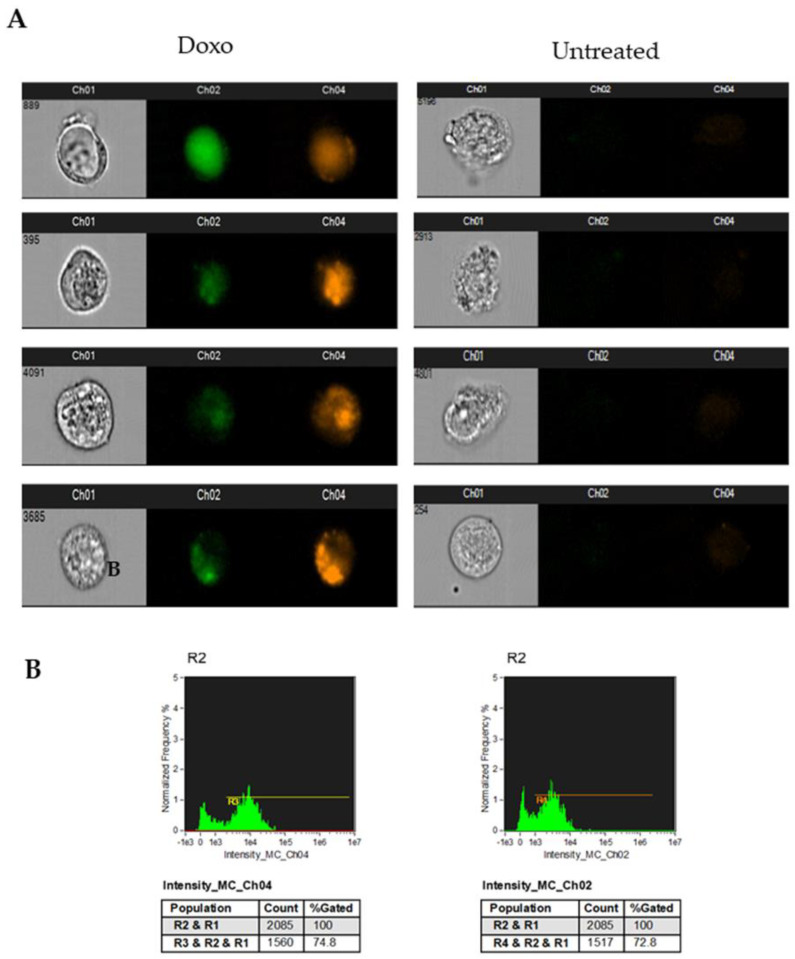
Analysis of doxorubicin fluorescence emission with ImageStream. MDA-MB-231 cells treated for 24 h with 1 µM doxorubicin were acquired using ImageStream: (**A**) representative images of treated cell brightfield and fluorescence detected in channel 1 (FITC and analogue fluorochromes) and channel 4 (propidium iodide and analogue fluorochromes) for representative cells treated with doxorubicin (Doxo) or untreated are shown; (**B**) histograms represent doxorubicin autofluorescence detected in channel 1 (FITC and analogue fluorochromes) and channel 4 (propidium iodide and analogue fluorochromes). Data are representative of three independent experiments.

In Figure 3, the ImageStream analysis of the doxorubicin-treated cells showed that anthracycline autofluorescence affected the channels excited by the 488 nm laser, which have more often been used to analyze apoptosis (Ch1 and Ch4 usually used to detect FITC-conjugated Annexin V and propidium iodide, respectively).

### 3.4. Analysis of Doxorubicin-Induced Apoptosis in Human Breast Cancer Models

**Figure 4:**
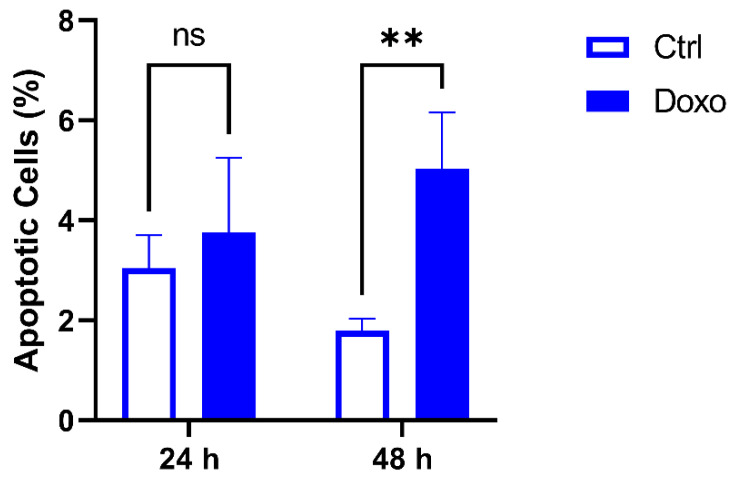
Apoptosis analysis using flow cytometry after staining with Annexin V-BV450. Histograms show the percentage of apoptotic MDA-MB-231 cells after the treatment with doxorubicin at 2.5 μM for 24 h or 48 h. Data are presented as the means ± SD of triplicate experiments. ** *p* < 0.01 vs. control (ns, not significant).

Considering previous results showing that anthracycline autofluorescence affects many channels of conventional flow cytometry platforms, the analysis of apoptosis or other markers through flow cytometry must consider such a phenomenon. Therefore, when apoptosis is measured by the detection of phosphatidylserine exposure, it would be more appropriate to consider detecting Annexin V (which binds phosphatidylserine) in some of the anthracycline non-affected channels. In this context, as shown above, the best channels to use are the ones excited by the 633 nm or 405 nm laser. An example of doxorubicin-treated MDA-MB-231 cells (24 and 48 h) stained with the BV450-conjugated Annexin V is reported in Figure S1. In addition, a statistically significant increase in Annexin V+ cells was observed after 48 h of treatment with doxorubicin (Figure 4). 

## 4. Discussion

Recent efforts in the precision medicine field allowed the development of patient-derived in vitro assays with the potential to predict treatment response [32]. The establishment of precision medicine platforms represents a future challenge for identifying effective drugs at a single-patient level [33,34]. The development of specific in vitro strategies for improved personalized cancer treatment has the potential to ameliorate patient management and reduce the cost of cancer care [34]. The first step for the optimization of novel in vitro approaches is the assessment of solid protocols to study the drug effects on specific cellular models. In this context, in vitro studies on anthracycline derivatives may have significant relevance, given that anthracyclines are widely employed in clinical practice [14,15,16]. 

It is worth noting that anthracycline derivatives are characterized by a typical red fluorescence emission [35]. These optical properties of anthracycline derivatives were successfully exploited for the investigation of drug dynamics in carcinoma treatments [36], to analyze the localization of anthracyclines in the lipid bilayers, and to assess the interaction of those drugs with the DNA, as well as other macromolecules within the target cells [37]. Furthermore, the study of the anthracycline intrinsic fluorescence was used in the imaging studies of living cells [38] and to track anthracyclines in human body fluids [39,40,41]. In such a context, when anthracycline autofluorescence is tracked by flow cytometry, our data reported that the best channels for doxorubicin and epirubicin acquisition were PerCP-Cy5.5/PC5. Besides the utility in monitoring anthracycline autofluorescence in the above-mentioned experimental settings, anthracycline autofluorescence should be carefully evaluated when cancer cells are treated with this class of drugs and then stained with fluorescent reagents and/or analyzed with fluorescence-based techniques. Cell autofluorescence is, in fact, an undesired source of background interference with the signal coming from dim fluorophores and/or low abundant markers [42,43]. Notably, the possibility to subtract the background produced by the autofluorescence of the used reagents has been largely underlined by the advent of spectral flow cytometry [44].

However, to the best of our knowledge, there are no published papers establishing the best practice to study anthracycline-induced cytotoxic effects through conventional flow cytometry. In the present work, we investigated the best markers or combination of markers to study the in vitro effects of anthracyclines using flow cytometry. In this regard, we observed that all the channels excited by the 488 nm laser were largely affected by the anthracycline autofluorescence after the treatment of breast cancer cells with the most widely used anthracyclines (doxorubicin and epirubicin). This effect was time-dependent. Furthermore, we reported that probes/fluorochromes excited by the red laser (633 nm), the ones excited by the violet laser (405 nm), and those emitting at lower wavelengths are recommended when anthracyclines are investigated in in vitro studies employing flow cytometry. 

Taken together, our findings may have two main implications. First, it is highly recommended to carefully use propidium iodide (emitting where the doxorubicin/epirubicin peaks of emissions are registered) when anthracycline-treated cells are analyzed using common and traditional flow cytometry platforms. Secondly, anthracycline autofluorescence should be considered when applying a technique or protocol involving the use of fluorescent probes or fluorochromes to study the in vitro effects of anthracyclines (i.e., immunofluorescence). In this respect, we observed that the percentage of apoptotic cells in the samples treated with doxorubicin revealed robust and highly reproducible values (low SD values) when the staining of the phosphatidylserine was performed with an appropriate reagent (Annexin V-BV450 conjugated). 

## 5. Conclusions

Altogether, our data established a set of recommendations that enable the development of optimized flow cytometry settings when the in vitro studies of anthracycline effects are carried out. These recommendations maximize the ability to reliably distinguish the positive and negative populations of anthracycline-treated cells. By these findings, the formulation of a consensus regarding the utility of flow cytometry for the analysis of anthracycline-treated samples may be established, unveiling a new perspective on the use of those in vitro tests for the further implementation of precision medicine strategies.

## Figures and Tables

**Table 1:** Signal-to-noise ratio (SNR) values.

	Channel	Filters	SNR Doxorubicin	SNR Epirubicin
**FACS CantoII**	FITC	502 LP 530/30	8.45	12.92
	PE	556 LP 585/42	94.80	164.06
	PerCP-Cy5-5	655 LP 670 LP	171.29	412.03
	PE-Cy7	735 LP 780/60	148.60	391.92
	APC	660/20	2.31	2.12
	APC-Cy7	735 LP 780/60	2.52	2.58
	Pacific Blue	450/50	1.08	1.01
	AmCyan	502 LP 510/50	1.14	1.14
**FACS Verse**	FITC	507 LP 527/32	10.15	12.89
	PE	560 LP 586/42	116.96	171.54
	PerCP-Cy5-5	665 LP 700/54	219.69	459.87
	PE-Cy7	752 LP 783/56	190.82	411.52
	APC	610/610 660/10	2.17	2.22
	APC-Cy7	752 LP 783/56	2.36	2.43
	V450	448/45 448/45	1.25	1.16
	V500	500 LP 528/45	2.71	3.08
**CytoFLEX**	FITC	525/40	11.27	14.82
	PE	585/42	105.67	156.59
	ECD	610/20	179.03	323.83
	PC5.5	690/50	219.21	469.39
	PC7	780/60	188.31	446.30
	APC	660/10	2.46	2.41
	APC-A700	712/25	2.47	2.54
	APC-A750	780/60	2.51	2.75
	PB450	450/45	1.29	1.24
	KO525	525/40	2.20	2.45
	Violet 610	610/20	48.19	90.47
	Violet 780	780/60	47.78	122.05

## Data Availability

Data are available upon appropriate request.

## Supplementary Materials

The following supporting information can be downloaded at: https://www.mdpi.com/article/10.3390/cimb45010013/s1, Figure S1: Gating strategy of Annexin V MDA-MB-231 cells; Figure S2: Analysis of Doxorubicin fluorescence emission by a FACSCanto II; Figure S3: Analysis of Doxorubicin fluorescence emission by a CytoFLEX; Figure S4: Analysis of Epirubicin fluorescence emission by a FACSCanto II; Figure S5: Analysis of Epirubicin fluorescence emission by a CytoFLEX.

## References

[1] Zoli W., Ricotti L., Tesei A., Barzanti F., Amadori D. (2001). In Vitro Preclinical Models for a Rational Design of Chemotherapy Combinations in Human Tumors. Crit. Rev. Oncol. Hematol..

[2] Di Tomo P., Alessio N., Falone S., Pietrangelo L., Lanuti P., Cordone V., Santini S.J., Di Pietrantonio N., Marchisio M., Protasi F. (2021). Endothelial Cells from Umbilical Cord of Women Affected by Gestational Diabetes: A Suitable in Vitro Model to Study Mechanisms of Early Vascular Senescence in Diabetes. FASEB J..

[3] Lanuti P., Bertagnolo V., Pierdomenico L., Bascelli A., Santavenere E., Alinari L., Capitani S., Miscia S., Marchisio M. (2009). Enhancement of TRAIL Cytotoxicity by AG-490 in Human ALL Cells Is Characterized by Downregulation of CIAP-1 and CIAP-2 through Inhibition of Jak2/Stat3. Cell Res..

[4] Lanuti P., Bertagnolo V., Gaspari A.R., Ciccocioppo F., Pierdomenico L., Bascelli A., Sabatino G., Miscia S., Marchisio M. (2006). Parallel Regulation of PKC-Alpha and PKC-Delta Characterizes the Occurrence of Erythroid Differentiation from Human Primary Hematopoietic Progenitors. Exp. Hematol..

[5] Lanuti P., Marchisio M., Cantilena S., Paludi M., Bascelli A., Gaspari A.R., Grifone G., Centurione M.A., Papa S., Di Pietro R. (2006). A Flow Cytometry Procedure for Simultaneous Characterization of Cell DNA Content and Expression of Intracellular Protein Kinase C-Zeta. J. Immunol. Methods.

[6] Bologna G., Lanuti P., D’Ambrosio P., Tonucci L., Pierdomenico L., D’Emilio C., Celli N., Marchisio M., D’Alessandro N., Santavenere E. (2014). Water-Soluble Platinum Phthalocyanines as Potential Antitumor Agents. Biometals.

[7] Di Tomo P., Pipino C., Lanuti P., Morabito C., Pierdomenico L., Sirolli V., Bonomini M., Miscia S., Mariggiò M.A., Marchisio M. (2013). Calcium Sensing Receptor Expression in Ovine Amniotic Fluid Mesenchymal Stem Cells and the Potential Role of R-568 during Osteogenic Differentiation. PLoS ONE.

[8] D’Alimonte I., Nargi E., Zuccarini M., Lanuti P., Di Iorio P., Giuliani P., Ricci-Vitiani L., Pallini R., Caciagli F., Ciccarelli R. (2015). Potentiation of Temozolomide Antitumor Effect by Purine Receptor Ligands Able to Restrain the in Vitro Growth of Human Glioblastoma Stem Cells. Purinergic Signal.

[9] Veschi S., De Lellis L., Florio R., Lanuti P., Massucci A., Tinari N., De Tursi M., di Sebastiano P., Marchisio M., Natoli C. (2018). Effects of Repurposed Drug Candidates Nitroxoline and Nelfinavir as Single Agents or in Combination with Erlotinib in Pancreatic Cancer Cells. J. Exp. Clin. Cancer Res..

[10] Di Tomo P., Lanuti P., Di Pietro N., Baldassarre M.P.A., Marchisio M., Pandolfi A., Consoli A., Formoso G. (2017). Liraglutide Mitigates TNF-α Induced pro-Atherogenic Changes and Microvesicle Release in HUVEC from Diabetic Women. Diabetes Metab. Res. Rev..

[11] Codagnone M., Recchiuti A., Lanuti P., Pierdomenico A.M., Cianci E., Patruno S., Mari V.C., Simiele F., Di Tomo P., Pandolfi A. (2017). Lipoxin A4 Stimulates Endothelial MiR-126-5p Expression and Its Transfer via Microvesicles. FASEB J..

[12] Minotti G., Menna P., Salvatorelli E., Cairo G., Gianni L. (2004). Anthracyclines: Molecular Advances and Pharmacologic Developments in Antitumor Activity and Cardiotoxicity. Pharmacol. Rev..

[13] McGowan J.V., Chung R., Maulik A., Piotrowska I., Walker J.M., Yellon D.M. (2017). Anthracycline Chemotherapy and Cardiotoxicity. Cardiovasc. Drugs Ther..

[14] Megías-Vericat J.E., Martínez-Cuadrón D., Sanz M.Á., Poveda J.L., Montesinos P. (2019). Daunorubicin and Cytarabine for Certain Types of Poor-Prognosis Acute Myeloid Leukemia: A Systematic Literature Review. Expert Rev. Clin. Pharmacol..

[15] Antolín S., Acea B., Albaina L., Concha Á., Santiago P., García-Caballero T., Mosquera J.J., Varela J.R., Soler R., Calvo L. (2019). Primary Systemic Therapy in HER2-Positive Operable Breast Cancer Using Trastuzumab and Chemotherapy: Efficacy Data, Cardiotoxicity and Long-Term Follow-up in 142 Patients Diagnosed from 2005 to 2016 at a Single Institution. Breast Cancer Dove Med. Press.

[16] Meyer M., Seetharam M. (2019). First-Line Therapy for Metastatic Soft Tissue Sarcoma. Curr. Treat. Options Oncol..

[17] Singal P.K., Iliskovic N. (1998). Doxorubicin-Induced Cardiomyopathy. N. Engl. J. Med..

[18] Salvatorelli E., Guarnieri S., Menna P., Liberi G., Calafiore A.M., Mariggiò M.A., Mordente A., Gianni L., Minotti G. (2006). Defective One- or Two-Electron Reduction of the Anticancer Anthracycline Epirubicin in Human Heart. Relative Importance of Vesicular Sequestration and Impaired Efficiency of Electron Addition. J. Biol. Chem..

[19] Gewirtz D.A. (1999). A Critical Evaluation of the Mechanisms of Action Proposed for the Antitumor Effects of the Anthracycline Antibiotics Adriamycin and Daunorubicin. Biochem. Pharmacol..

[20] Venkatesh P., Kasi A. (2022). Anthracyclines.

[21] Christidi E., Brunham L.R. (2021). Regulated Cell Death Pathways in Doxorubicin-Induced Cardiotoxicity. Cell Death Discov..

[22] Wlodkowic D., Skommer J., Darzynkiewicz Z. (2009). Flow Cytometry-Based Apoptosis Detection. Methods Mol. Biol..

[23] Martin J.C., Sims J.R., Gupta A., Hagoel T.J., Gao L., Lynch M.L., Woloszynska A., Melendy T., Kane J.F., Kuechle J. (2022). CDC7 Kinase (DDK) Inhibition Disrupts DNA Replication Leading to Mitotic Catastrophe in Ewing Sarcoma. Cell Death Discov..

[24] Huigsloot M., Tijdens I.B., Mulder G.J., van de Water B. (2002). Differential Regulation of Doxorubicin-Induced Mitochondrial Dysfunction and Apoptosis by Bcl-2 in Mammary Adenocarcinoma (MTLn3) Cells. J. Biol. Chem..

[25] Wilson C.O., Gisvold O., Block J.H., Beale J.M., Wilson C.O. (2004). Wilson and Gisvold’s Textbook of Organic Medicinal and Pharmaceutical Chemistry.

[26] Kauffman M.K., Kauffman M.E., Zhu H., Jia Z., Li Y.R. (2016). Fluorescence-Based Assays for Measuring Doxorubicin in Biological Systems. React Oxyg Species (Apex NC).

[27] Dine T., Brunet C., Luyckx M., Cazin M., Gosselin P., Cazin J.L. (1990). Rapid Quantitative Determination of Epirubicin and Its Metabolites in Plasma Using High Performance Liquid Chromatography and Fluorescence Detection. Biomed. Chromatogr..

[28] Guo B., Tam A., Santi S.A., Parissenti A.M. (2016). Role of Autophagy and Lysosomal Drug Sequestration in Acquired Resistance to Doxorubicin in MCF-7 Cells. BMC Cancer.

[29] Lovitt C.J., Shelper T.B., Avery V.M. (2018). Doxorubicin Resistance in Breast Cancer Cells Is Mediated by Extracellular Matrix Proteins. BMC Cancer.

[30] Chattopadhyay P.K., Hogerkorp C.-M., Roederer M. (2008). A Chromatic Explosion: The Development and Future of Multiparameter Flow Cytometry. Immunology.

[31] Lanuti P., Ciccocioppo F., Bonanni L., Marchisio M., Lachmann R., Tabet N., Pierdomenico L., Santavenere E., Catinella V., Iacone A. (2012). Amyloid-Specific T-Cells Differentiate Alzheimer’s Disease from Lewy Body Dementia. Neurobiol. Aging.

[32] Dumas M.-P., Xia S., Bear C.E., Ratjen F. (2021). Perspectives on the Translation of In-Vitro Studies to Precision Medicine in Cystic Fibrosis. EBioMedicine.

[33] Pauli C., Hopkins B.D., Prandi D., Shaw R., Fedrizzi T., Sboner A., Sailer V., Augello M., Puca L., Rosati R. (2017). Personalized In Vitro and In Vivo Cancer Models to Guide Precision Medicine. Cancer Discov..

[34] Schilsky R.L. (2010). Personalized Medicine in Oncology: The Future Is Now. Nat. Rev. Drug Discov..

[35] Krishan A., Ganapathi R. (1980). Laser Flow Cytometric Studies on the Intracellular Fluorescence of Anthracyclines. Cancer Res..

[36] Motlagh N.S.H., Parvin P., Ghasemi F., Atyabi F. (2016). Fluorescence Properties of Several Chemotherapy Drugs: Doxorubicin, Paclitaxel and Bleomycin. Biomed. Opt. Express.

[37] Karukstis K.K., Thompson E.H., Whiles J.A., Rosenfeld R.J. (1998). Deciphering the Fluorescence Signature of Daunomycin and Doxorubicin. Biophys. Chem..

[38] Dai X., Yue Z., Eccleston M.E., Swartling J., Slater N.K.H., Kaminski C.F. (2008). Fluorescence Intensity and Lifetime Imaging of Free and Micellar-Encapsulated Doxorubicin in Living Cells. Nanomedicine.

[39] Trevisan M.G., Poppi R.J. (2003). Determination of Doxorubicin in Human Plasma by Excitation–Emission Matrix Fluorescence and Multi-Way Analysis. Anal. Chim. Acta.

[40] Prakash J., Mishra A.K. (2014). Quantification of Doxorubicin in Biofluids Using White Light Excitation Fluorescence. J. Biophotonics.

[41] Martínez Ferreras F., Wolfbeis O.S., Gorris H.H. (2012). Dual Lifetime Referenced Fluorometry for the Determination of Doxorubicin in Urine. Anal. Chim. Acta.

[42] Hulspas R., O’Gorman M.R.G., Wood B.L., Gratama J.W., Sutherland D.R. (2009). Considerations for the Control of Background Fluorescence in Clinical Flow Cytometry. Cytom. B Clin. Cytom..

[43] Sebestyén Z., Nagy P., Horváth G., Vámosi G., Debets R., Gratama J.W., Alexander D.R., Szöllosi J. (2002). Long Wavelength Fluorophores and Cell-by-Cell Correction for Autofluorescence Significantly Improves the Accuracy of Flow Cytometric Energy Transfer Measurements on a Dual-Laser Benchtop Flow Cytometer. Cytometry.

[44] Sanders C.K., Mourant J.R. (2013). Advantages of Full Spectrum Flow Cytometry. J. Biomed. Opt..

