# Supplementary material for: Flow Cytometry Detection of Anthracycline-Treated Breast Cancer Cells: An Optimized Protocol
Source: Curr Issues Mol Biol. 2022 Dec 28;45(1):164–74. doi: 10.3390/cimb45010013 (PMC9857732; doi:10.3390/cimb45010013)
Supplement: Supplementary file 1 [file cimb-45-00013-s001.zip › Supplement material.pdf]

Staining of DOXO treated MDA-MB-231 cells

-MDA-MB-231 cells were counted with a Burker chamber and seeded in 6-well plates (2 x 105 cells/well) in triplicate;

-Cells were treated with 2.5 µM Doxorubicin for 24 or 48h;

-After treatment, apoptosis was measured saving the supernatants before detaching cells by trypsinization cells were detached by trypsinization and washed by centrifugation at 400g for 10 minutes once in PBS (ThermoFisher Scientific, Gibco; Waltham, MA, USA).

-The supernatant was discarded, and the cell pellet was resuspended at a concentration of 5 x 10^5 cells/mL in Binding Buffer 1X (Becton Dickinson -BD- Biosciences, La Jolla, CA, USA).

-Samples were centrifuged at 400g for 10 minutes

-Samples were stained, using 5 µL of Annexin V-BV450 (BD Biosciences, Cod. 560506) and incubated for 15 minutes at room temperature in the dark.

- Before the acquisition, 300 uL of Binding Buffer 1X was added;

-For each sample, 20,000 events were acquired using a FACSVerse flow cytometer (BD Biosciences);

-The MDA-MB-231 cells were identified in the FSC-A/SSC-A dot plot;

-The doublets were excluded in the FSC-A/FSC-H plot ;

-Apoptotic cells were identified for their positivity to Annexin V.
