# Supplementary material for: Flow Cytometry Detection of Anthracycline-Treated Breast Cancer Cells: An Optimized Protocol
Source: Curr Issues Mol Biol. 2022 Dec 28;45(1):164–74. doi: 10.3390/cimb45010013 (PMC9857732; doi:10.3390/cimb45010013)
Supplement: Supplementary file 1 [file cimb-45-00013-s001.zip › Supplementary figures.pdf]

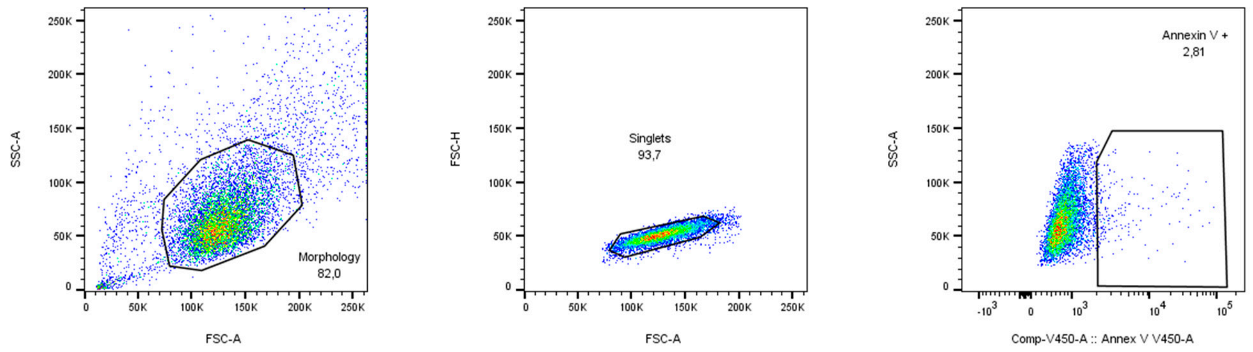

Figure S1. **Gating strategy of Annexin V MDA-MB-231 cells**. MDA-MB-231 cells were selected in a FCS-A/SSC-A gate; next doublets were excluded (FSC-A/FSC-H) and Annexin V+ cells were gated (Annexin V-A/SSC-A).

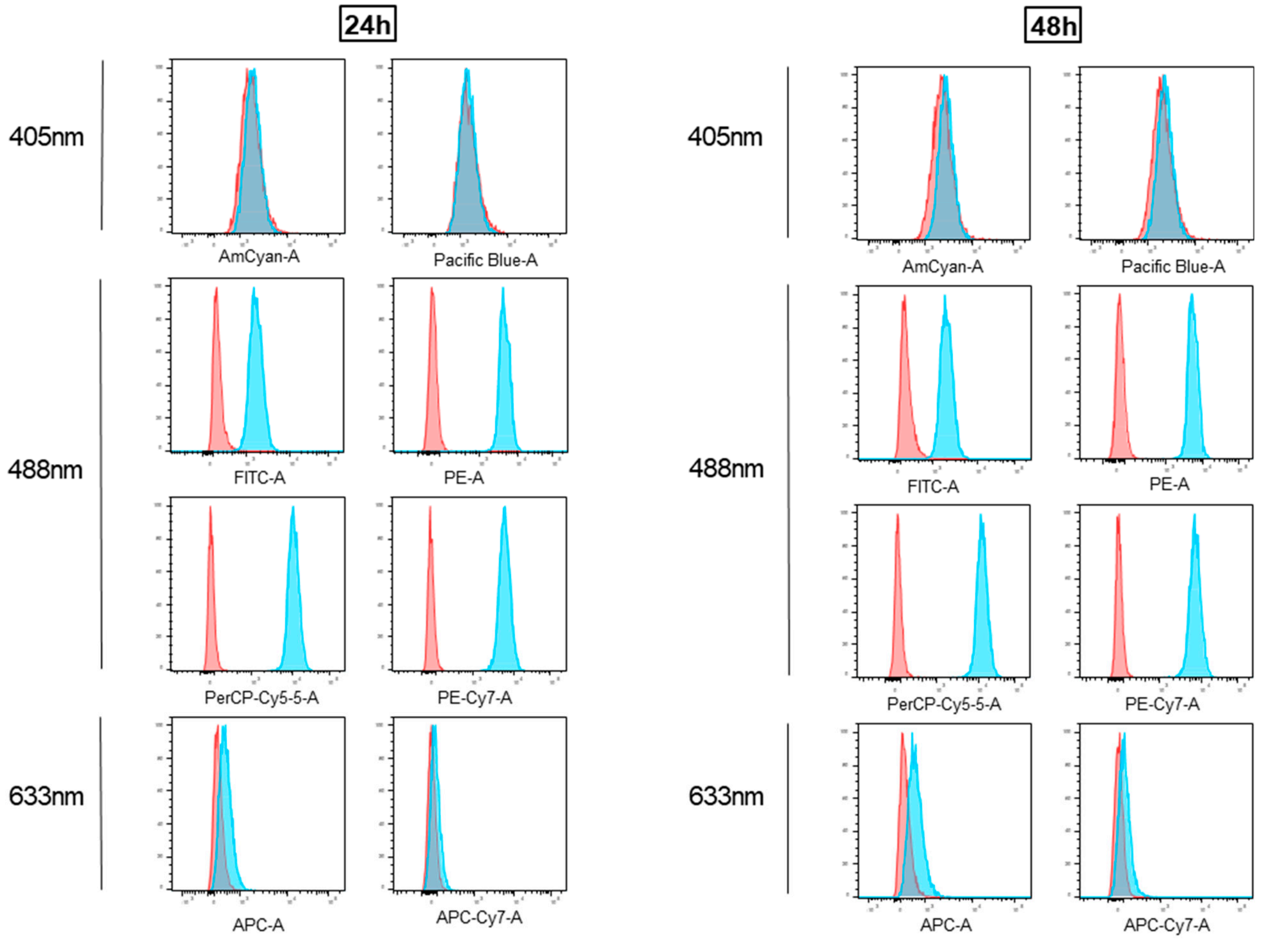

Figure S2. Analysis of Doxorubicin fluorescence emission by a FACSCanto II. Cells treated for 24 and 48h with 1µM (**Panel A**) or 2.5 µM (**Panel B**) Doxorubicin (Doxo) were acquired by flow cytometry and shown as blue histograms on every channel of a conventional instrument BD FACSCanto II, equipped with three lasers (488nm, 633nm and 405nm). Overlayed red histograms show the related untreated samples. Data are representative of three separate experiments.

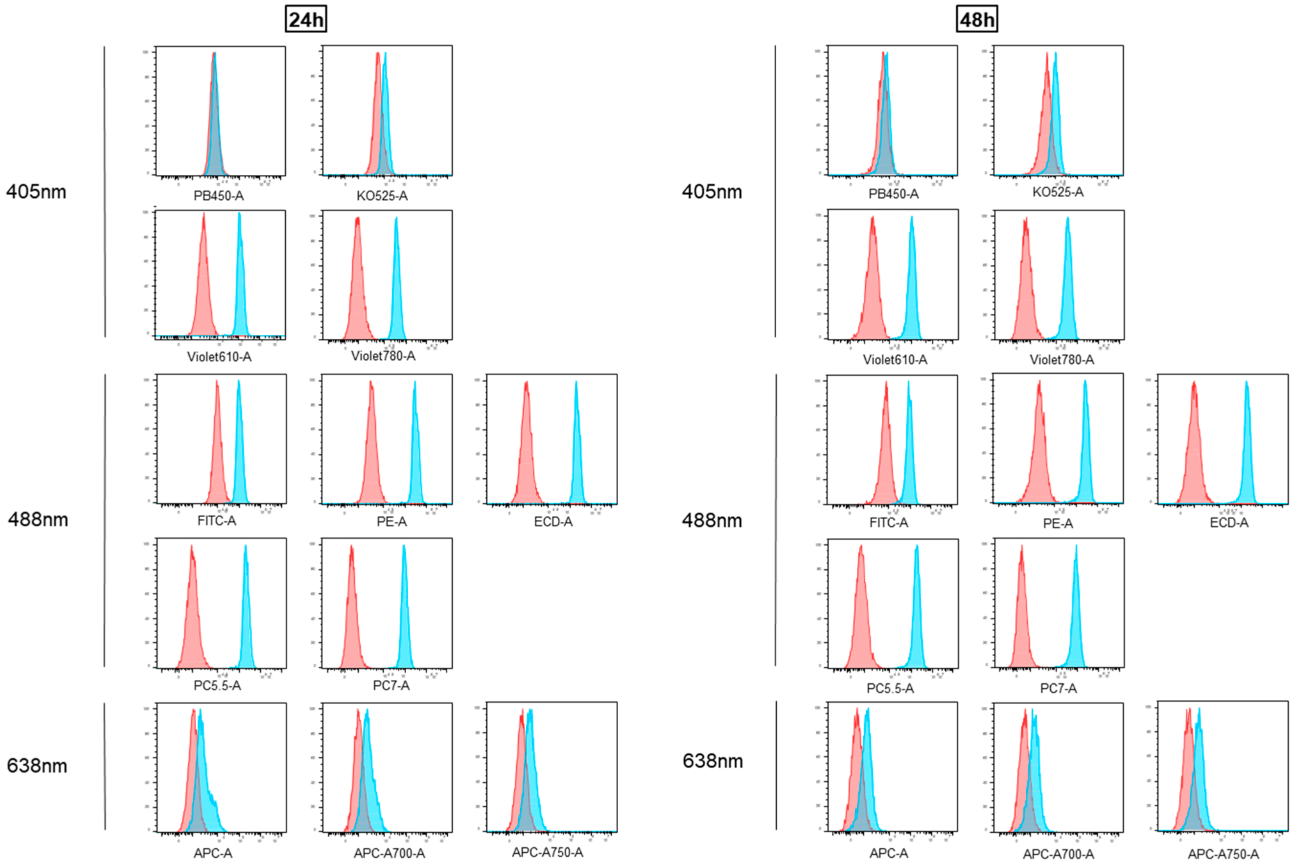

Figure S3. Analysis of Doxorubicin fluorescence emission by a CytoFLEX. Cells treated for 24 and 48h with 1µM (**Panel A**) or 2.5 µM (**Panel B**) Doxorubicin (Doxo) were acquired by flow cytometry and shown as blue histograms on every channel of a conventional instrument BC CytoFLEX, equipped with three lasers (488nm, 633nm and 405nm). Overlayed red histograms show the related untreated samples. Data are representative of three separate experiments.

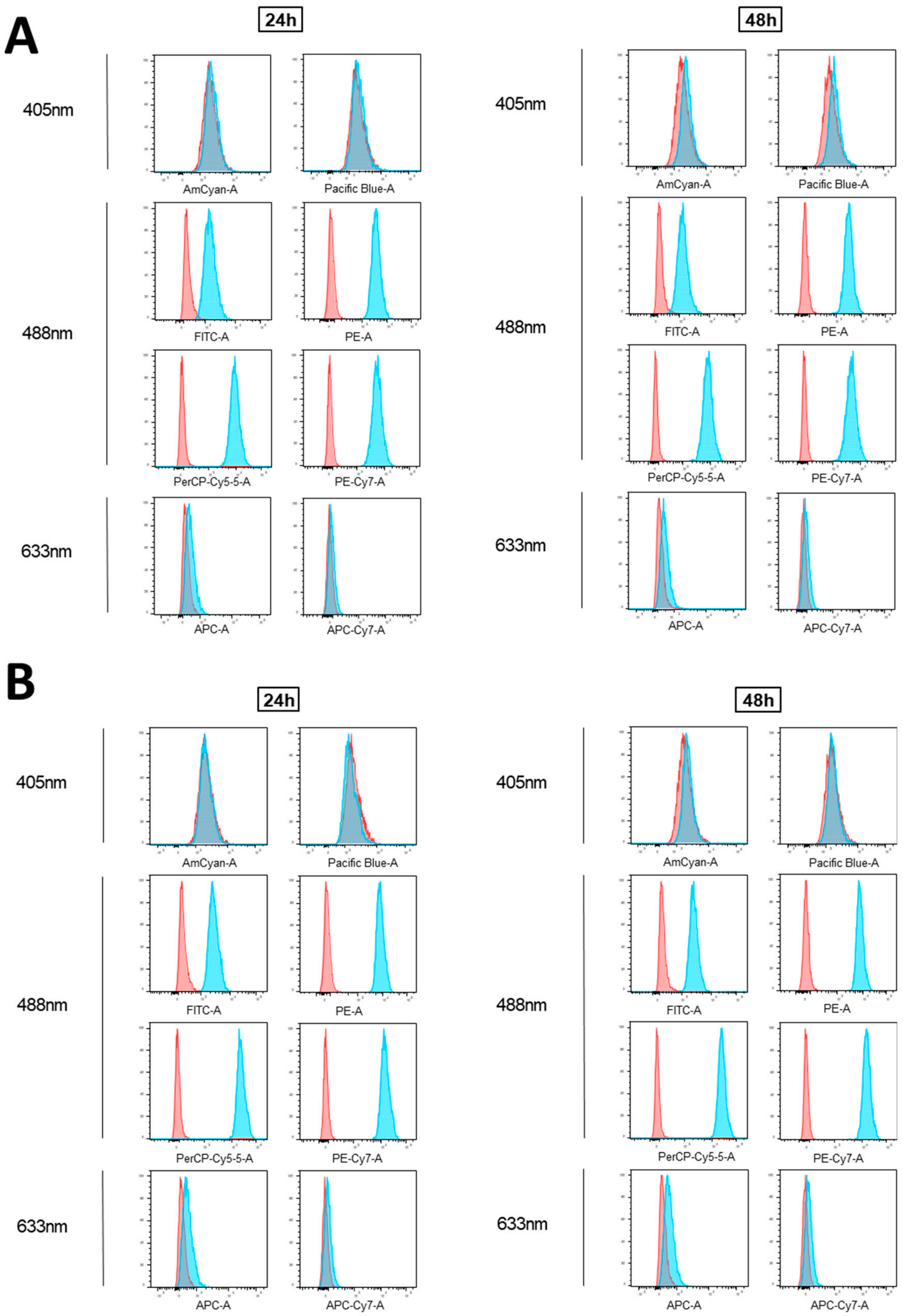
A
24h
48h
405nm
AmCyan-A
Pacific Blue-A
488nm
FITC-A
PE-A
PerCP-Cy5-5-A
PE-Cy7-A
633nm
APC-A
APC-Cy7-A
B
24h
48h
405nm
AmCyan-A
Pacific Blue-A
488nm
FITC-A
PE-A
PerCP-Cy5-5-A
PE-Cy7-A
633nm
APC-A
APC-Cy7-A

Figure S4. Analysis of Epirubicin fluorescence emission by a FACSCanto II. Cells treated for 24 and 48h with 1µM (**Panel A**) or 2.5 µM (**Panel B**) Epirubicin were acquired by flow cytometry and shown as blue histograms on every channel of a conventional instrument BD FACSCanto II, equipped with three lasers (488nm, 633nm and 405nm). Overlayed red histograms show the related untreated samples. Data are representative of three separate experiments.

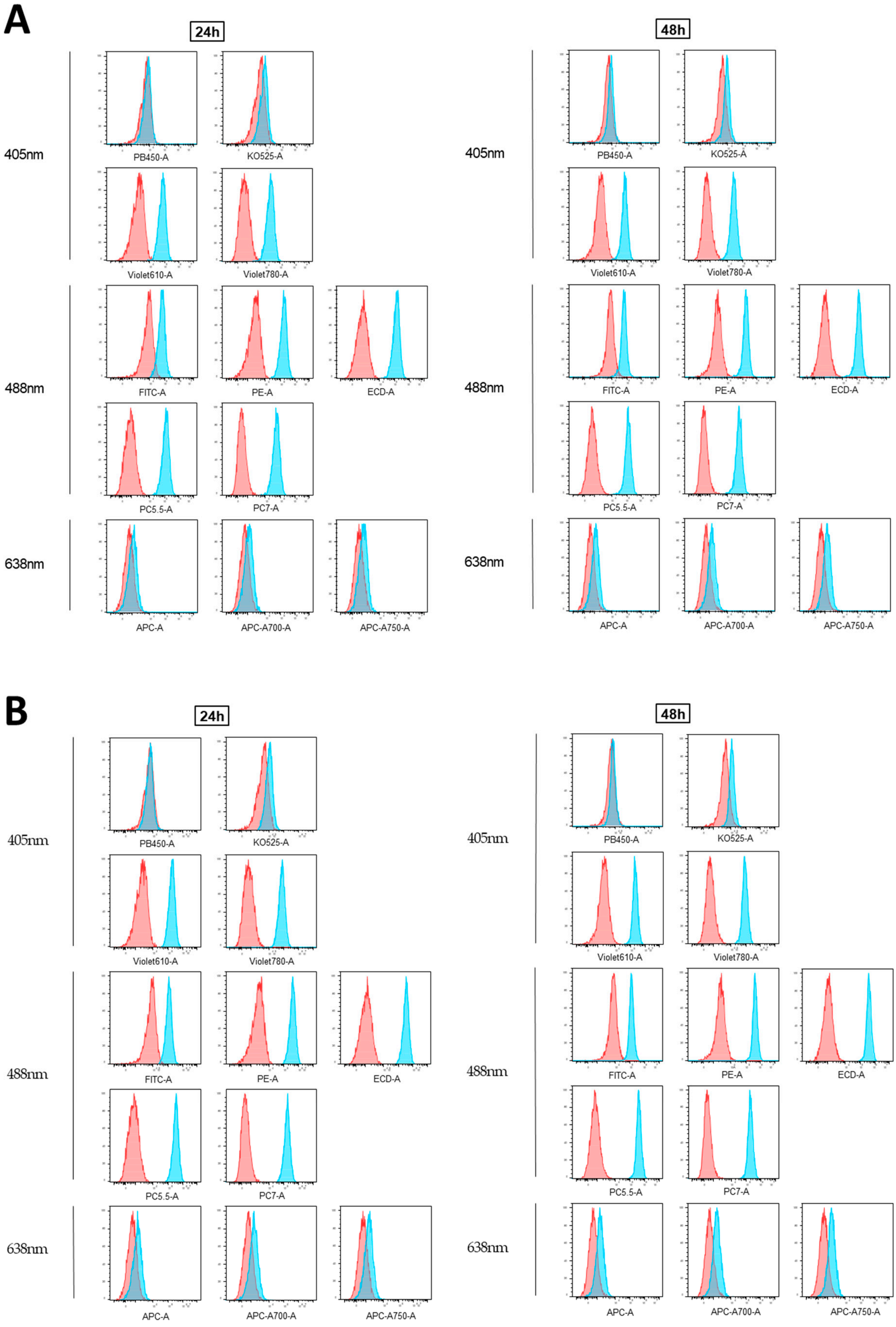
A
24h
48h
405nm
PB450-A
KO525-A
Violet610-A
Violet780-A
488nm
FITC-A
PE-A
ECD-A
PC5.5-A
PC7-A
638nm
APC-A
APC-A700-A
APC-A750-A
B
24h
48h
405nm
PB450-A
KO525-A
Violet610-A
Violet780-A
488nm
FITC-A
PE-A
ECD-A
PC5.5-A
PC7-A
638nm
APC-A
APC-A700-A
APC-A750-A

Figure S5. Analysis of Epirubicin fluorescence emission by a CytoFLEX. Cells treated for 24 and 48h with 1µM (**Panel A**) or 2.5 µM (**Panel B**) Epirubicin were acquired by flow cytometry and shown as blue histograms on every channel of a conventional instrument BC CytoFLEX, equipped with three lasers (488nm, 633nm and 405nm). Overlayed red histograms show the related untreated samples. Data are representative of three separate experiments.
